# Supplementary material for: Oceanic climate changes threaten the sustainability of Asia’s water tower
Source: Nature. 2023 Mar 1;615(7950):87–93. doi: 10.1038/s41586-022-05643-8 (PMC9977686; doi:10.1038/s41586-022-05643-8)
Supplement: Supplementary file 1 — This file contains Supplementary Notes 1 and 2 and Supplementary Figures 1–25. [file 41586_2022_5643_MOESM1_ESM.docx]

**Supplementary information for**

**Oceanic climate changes threaten the sustainability of Asia’s water tower**

**Supplementary file includes:**

Supplementary Notes 1-2

Supplementary Figures 1-25

**Supplementary Note 1 Determination of the number for the clustered trajectories**

Based on the determination standard^57^ for the optimal number of the clusters using k-means method, we initially determined the optimal number of the clusters for the trajectories is 3, and we further derived the 3 clusters of the trajectories from all trajectories (Supplementary Fig. 24). It suggests that there are only 3 eastward trajectories into TP without denoting the trajectory from the Indian Ocean (Supplementary Fig. 24). Although 3 is the optimal number of clusters, the result could not illustrate enough information about the transition path from the source regions to the TP. As for the criterion for determining the optimum number of clustered trajectories, we do not find relative standards in the handbook of the FLEXPARTv10.4 model and we also find various number of clusters used in other studies^48,52^. Thus, the optimum number of clusters is generally determined on a case-by-case basis.

In this study, we attempt to enrich the information of the trajectories and avoid far overlapping of the trajectories at same time. And we set the number of the clusters as 20, and derive 20 clustered trajectories (Supplementary Fig. 25). However, the 9^th^ trajectory has an abnormal turn from East Asia to TP, attributed to the disturbance induced by the zero-longitude centered map projection on the process of clustering trajectories. Since the rest of 19 trajectories are enough for illustrating the transition path from source regions to TP, thus, we finally only keep the 19 clustered trajectories in the main text.

**Supplementary Note 2 Notification for the selection of the index *i* in Eq. 19.**

In the Eq. 19, when k = 1, the index *i* is determined as (1, 4, 5, 6, 8) under SSP245 scenario and (1, 2, 4, 6, 8) under SSP585 scenario by the condition when the significant level *p* values distinctly large than 0.05 (see Supplementary Fig. 17). However, *p* values for the CMIP6 model 2 and 3 under SSP245 scenario are 0.069 and 0.067, which are close to 0.05 (Supplementary Fig. 17b-c). Thus, groups of $W_{1,s}$ determined by Eqs 19-20 under SSP245 scenario are further determined and compared with $Y_{1}$ when index *i* are (1, 2, 4, 5, 6, 8), (1, 3, 4, 5, 6, 8), (1, 2, 3, 4, 5, 6, 8) and (1, 4, 5, 6, 8). And the maximum correlation coefficient between $W_{1,s}$ and $Y_{1}$ (see Eq. 19) is 0.51 when index *i* is (1, 2, 4, 5, 6, 8). Thus, the index *i* is determined as (1, 2, 4, 5, 6, 8) when k = 1 under SSP245 scenario. When k = 2 or k = 3, the initially determined $W_{k,s}$ by Eqs. 19-20 is in good line with $Y_{k}$ (see Eq. 19) with correlation coefficients ranging from 0.58 to 0.93 (*p <* 0.05) (Supplementary Fig. 20). Thus, index *i* is not further modified when k = 2 or k = 3 in Eq. 19.

**
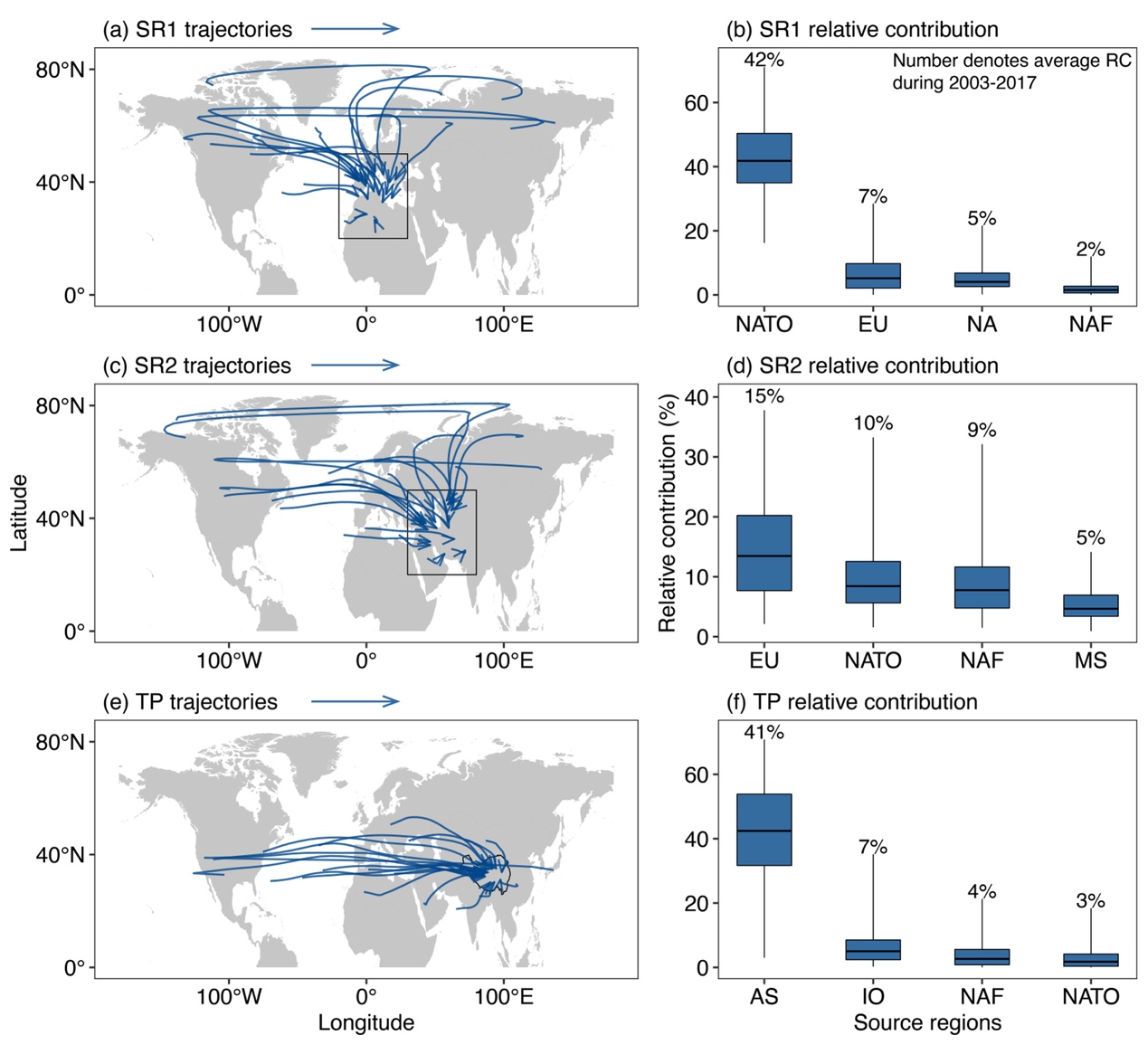
**

**Supplementary Figure 1. Backward trace analysis for SR1-2 and TP.** (a,c,e) Clustered trajectories into the SR1 (a), SR2 (c) and TP (e). (b,d,f) denote the monthly relative contributions of water vapor from main source regions to the SR1 (b), SR2 (d) and TP (f) when the on-route changes are disregarded. The boxplot margins mark the minimum, 25% quantile, median, 75% quantile and maximum value of the monthly relative contributions during 2003-2017. The continental world map data^31^ and map data of Tibet Plateau^32^ in panels **a**, **c** and **e** are acquired from public data source and plotted by R^33^. And results used to generate the figure are available in Zenodo^34^.


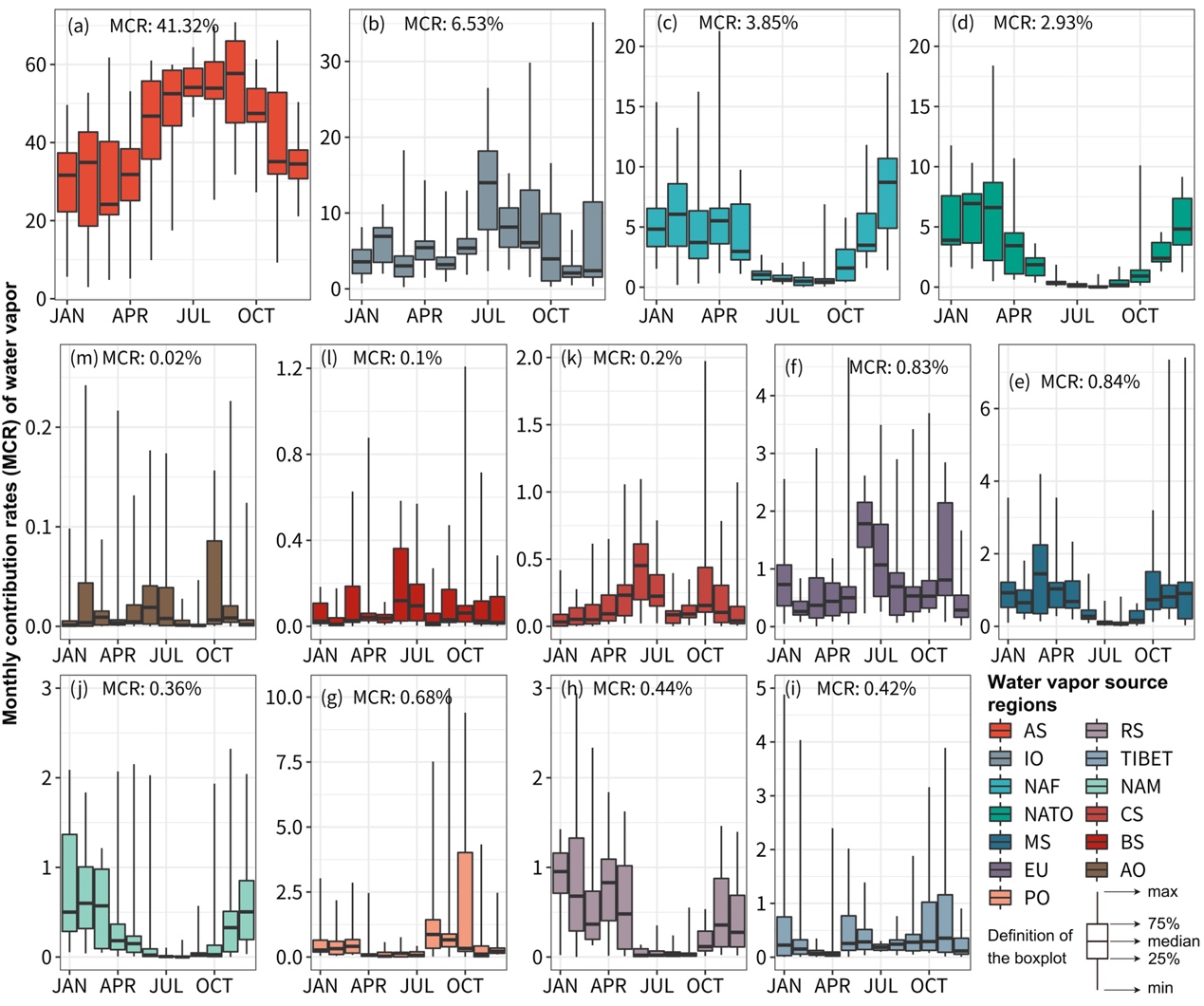


**Supplementary Figure 2. Monthly contribution rates accounting for on-route changes.** Panels “(a)” to “(m)” refer to the contribution rates of water vapor during 2003-2017 from Asia (AS), Indian Ocean (IO), North Africa (NAF), North Atlantic (NATO), Mediterranean Sea (MS), Europe (EU), Pacific Ocean (PO), Red Sea (RS), Tibet Plateau, North America (NAM), Caspian Sea (CS), Black Sea (BS), and Arctic Ocean (AO). And results used to generate the figure are available in Zenodo^34^.

**
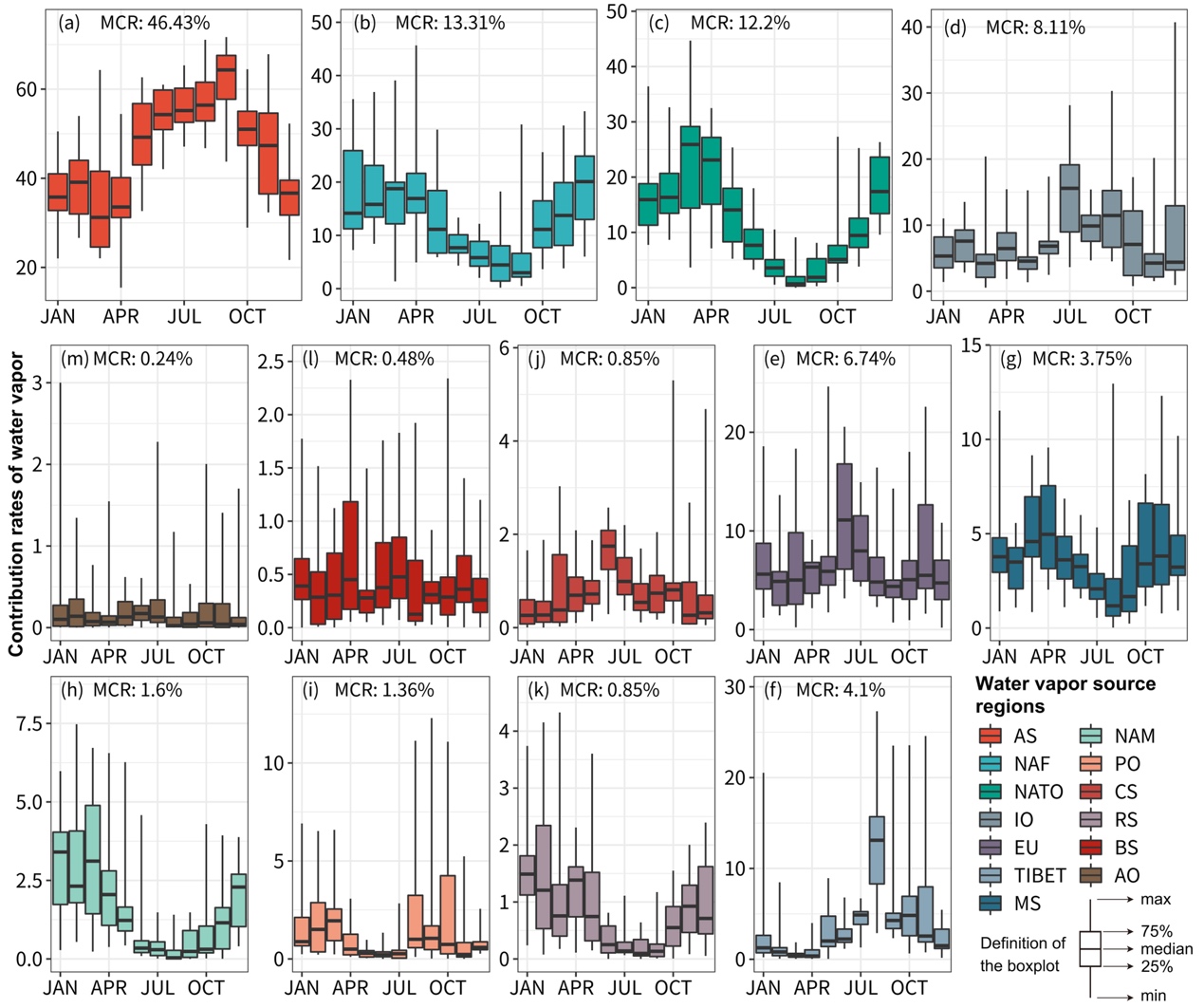
**

**Supplementary Figure 3. Monthly contribution rates disregarding on-route changes.** Panels “(a)” to “(m)” refer to the contribution rates during 2003-2017 from AS, IO, NAF, NATO, MS, EU, PO, RS, TP, NAM, CS, BS, and AO, respectively. And results used to generate the figure are available in Zenodo^34^.


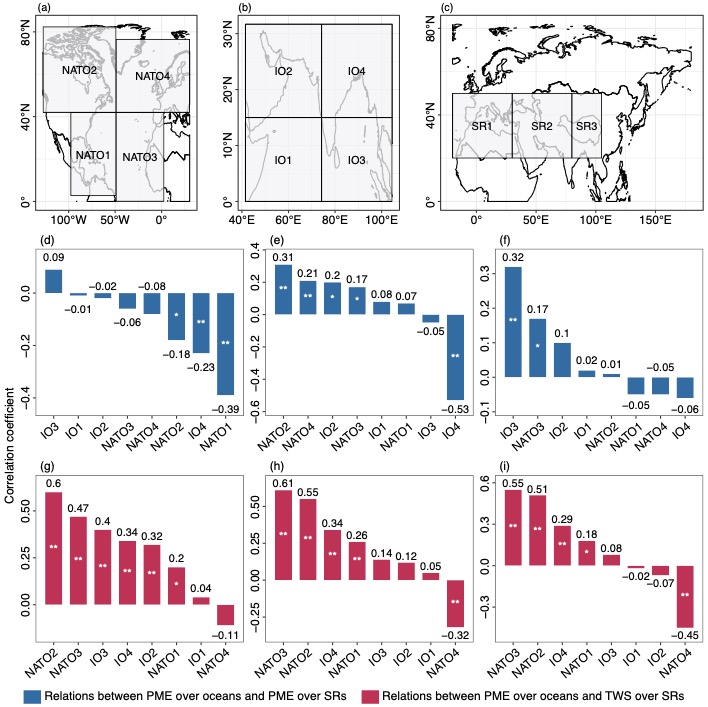


**Supplementary Figure 4. Relations between PME over oceans and PME and TWS over SRs.** (a-c) denote spatial locations of the subregions for North Atlantic (a), Indian Ocean (b) and mid-latitude Eurasia along water vapor trajectories to TP (c). (d-e) denote relations between PME over oceans and PME over SR1-3. (g-i) denote relations between PME over oceans and TWS over SR1-3. The continental world map data^31^ in panels **a-c** and the map data of Tibet Plateau^32^ in panel **c** are acquired from public data source and plotted by R^33^. And results used to generate the figure are available in Zenodo^34^.


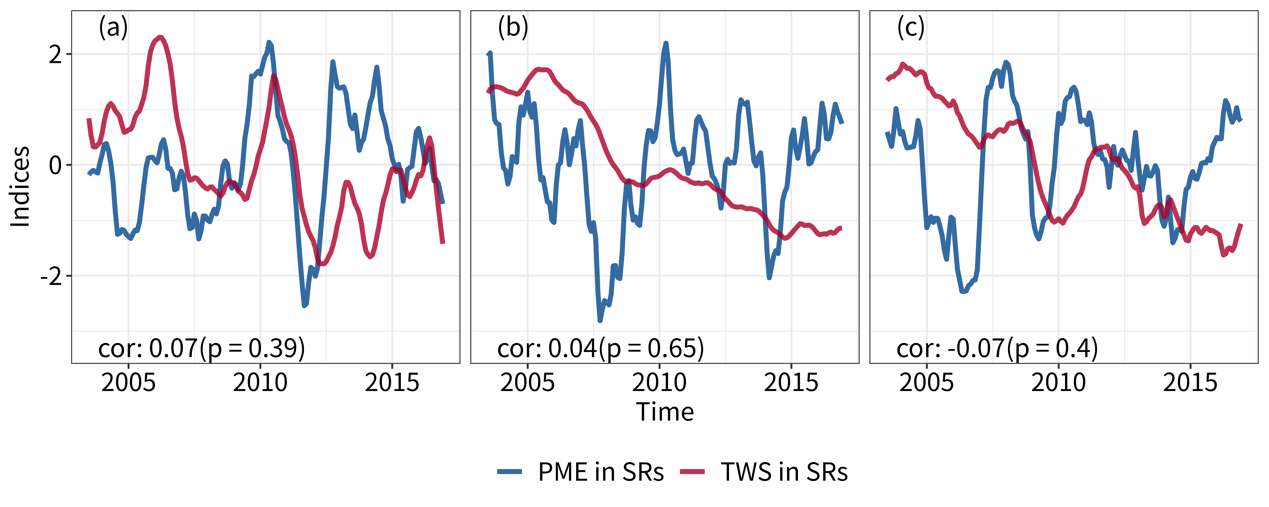


**Supplementary Figure 5. Relations between PMEs and TWSs in SR1-3.** (a-c) refer to the temporal variations in PMEs and TWSs in SR1-3, respectively. The “cor” in the plot refers to the correlation coefficient hereafter. And results used to generate the figure are available in Zenodo^34^.


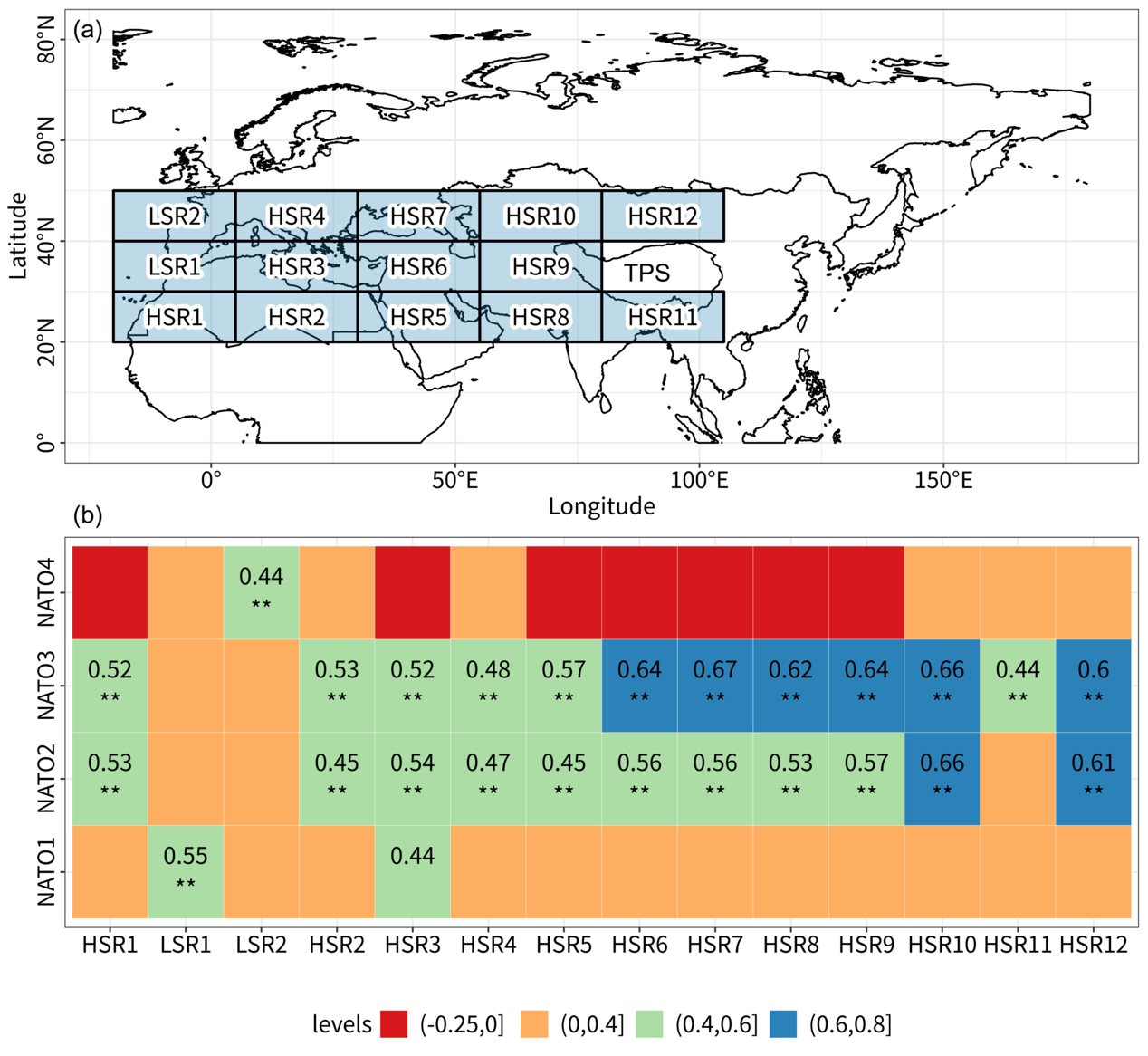


**Supplementary Figure 6. Cross correlations between PME over North Atlantic and TWS in lands.** (a) refers to the spatial locations of HSR1-12 and LSR1-2. (b) refers to the relations between TWSs in HSR1-12 and LSR1-2 and PMEs in regions of North Atlantic and Indian Ocean. The TPS in the plot refers to the central TP surface. The continental world map data^31^ and map data of Tibet Plateau^32^ in panel **a** are acquired from public data source and plotted by R^33^. Here, the sub-region where the cross-correlation coefficients >0.4 is defined as the HSR. Rest of sub-region is defined as the LSR. And results used to generate the figure are available in Zenodo^34^.


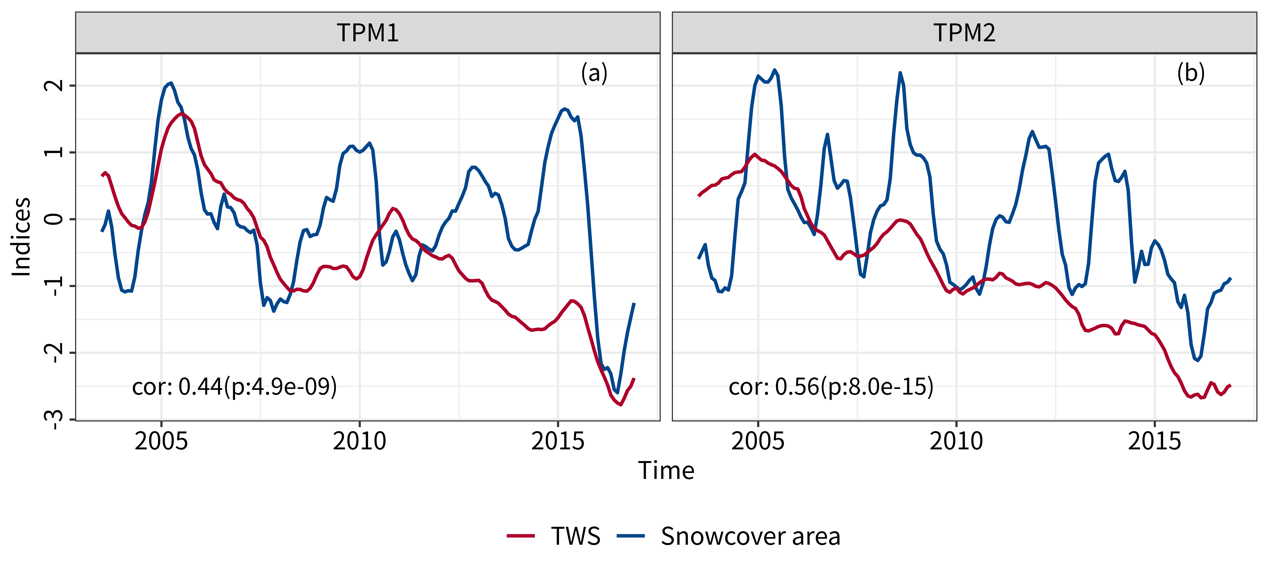


**Supplementary Figure 7. TWS and snow cover in the southern TP mountains.** (a-b) refer to the temporal variation in TWSs and snow cover in the southwest (TPM1) and southeast (TPM2) TP mountains. And results used to generate the figure are available in Zenodo^34^.


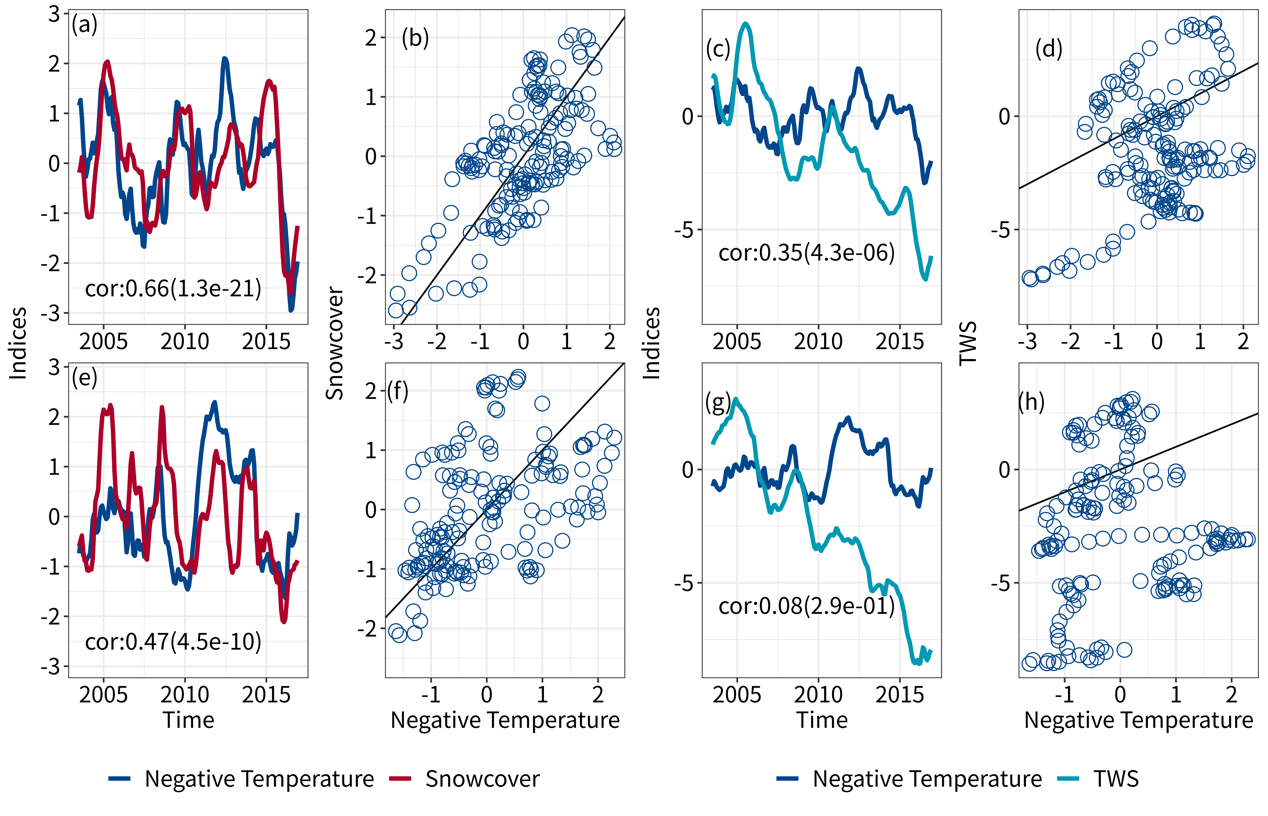


**Supplementary Figure 8. Relations between negative temperature and snow cover and TWS in the southern TP mountains.** (a-b, e-f) refer to the comparison between the snow cover and negative values of the temperature in the southwest (a-b) and southeast (e-f) TP mountain. (c-d, g-h) refer to the comparison between the TWS and negative values of the temperature in the southwest (c-d) and southeast (g-h) TP mountains. The negative temperature equals to -1 multiplies temperature. And results used to generate the figure are available in Zenodo^34^.


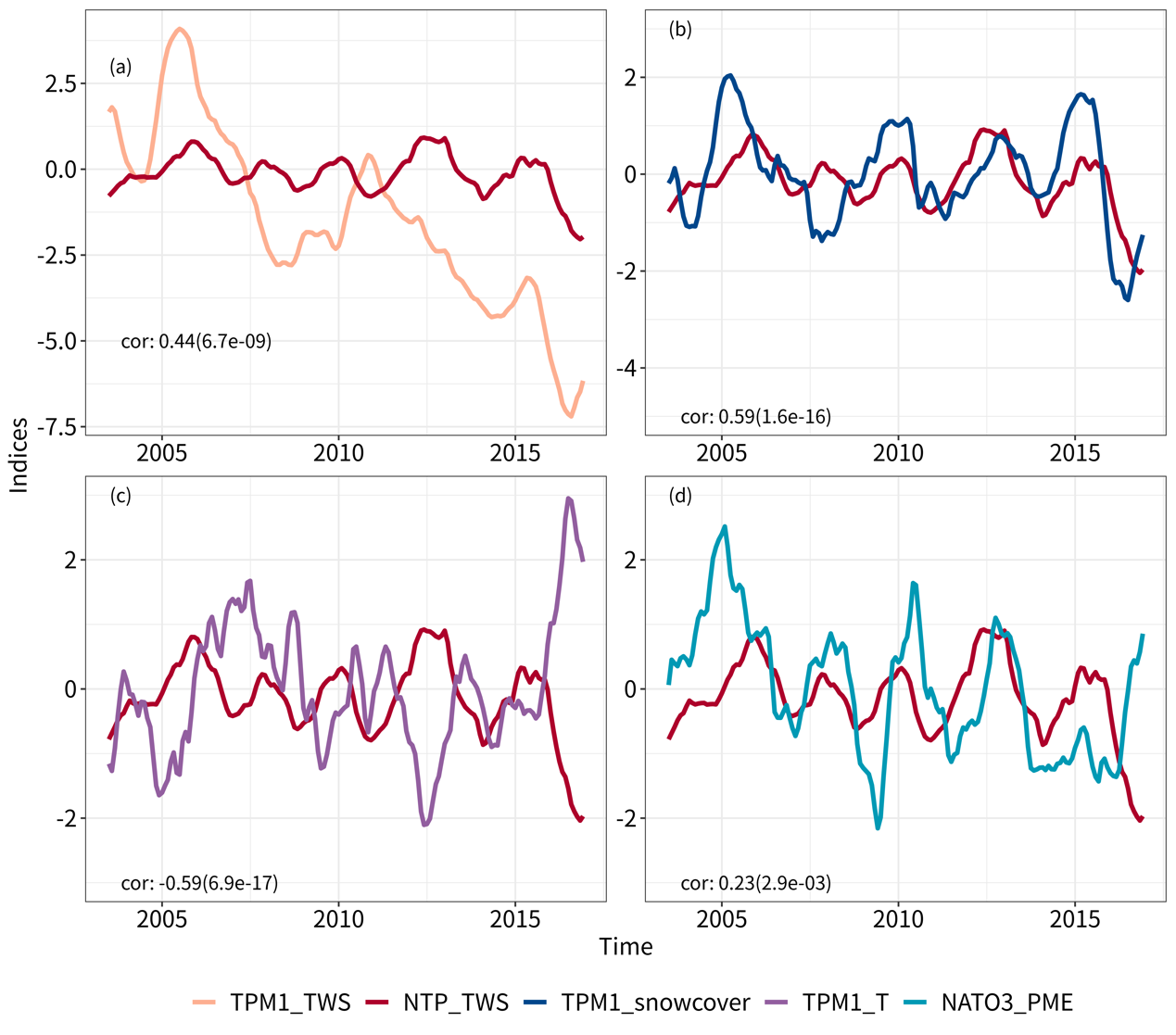


**Supplementary Figure 9. Comparison between TWS in the north TP and other indices in TPM1.** (a-c) refers to the temporal variations in TWS (a), snow cover area (b), T (c) in the southwest TP mountains (TPM1) and TWS in the north TP (NTP). (d) refers to the temporal variation in PME in southeast North Atlantic (NATO3) and TWS in the north TP (NTP). The “cor” denotes relations between two indices in each panel. And results used to generate the figure are available in Zenodo^34^.


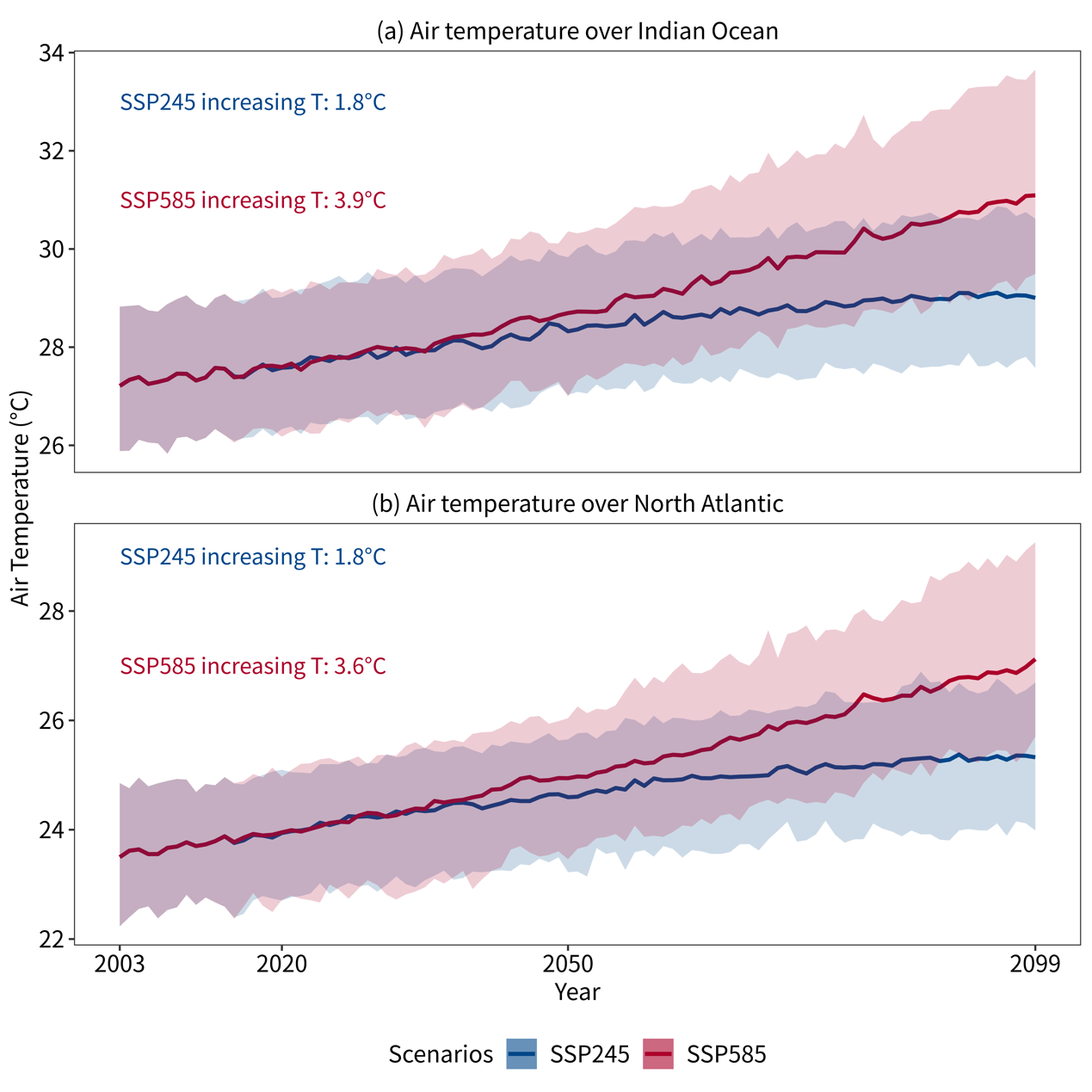


**Supplementary Figure 10. Air temperature over oceans.** (a) refers to the variation in the regional average air temperature over the Indian Ocean. (b) refers to the variation in the regional average air temperature over the south (including southeast and southwest) North Atlantic. And results used to generate the figure are available in Zenodo^34^.


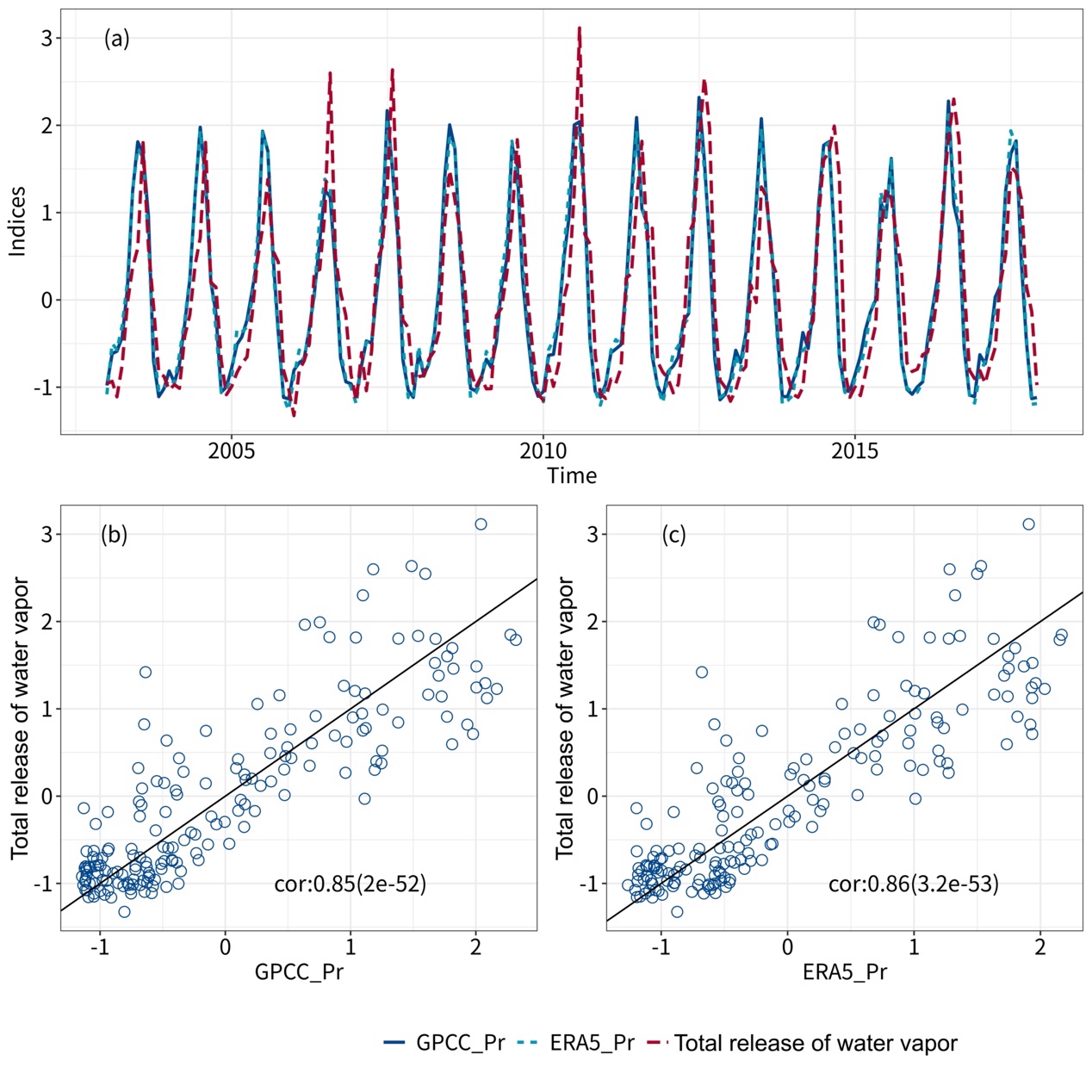


**Supplementary Figure 11. Comparisons between simulated total water vapor release and the GPCC and ERA5 precipitation in TP.** (a) refers to the temporal evolutions in the standardized GPCC and ERA5 precipitation, and the standardized total release of water vapor in the TP during 2003-2017. (b-c) refers to the comparison between the standardized GPCC (b) and ERA5 (c) precipitation, and the total release of water vapor in the TP. GPCC_Pr and ERA5_Pr denote GPCC and ERA5 precipitation here. And results used to generate the figure are available in Zenodo^34^.


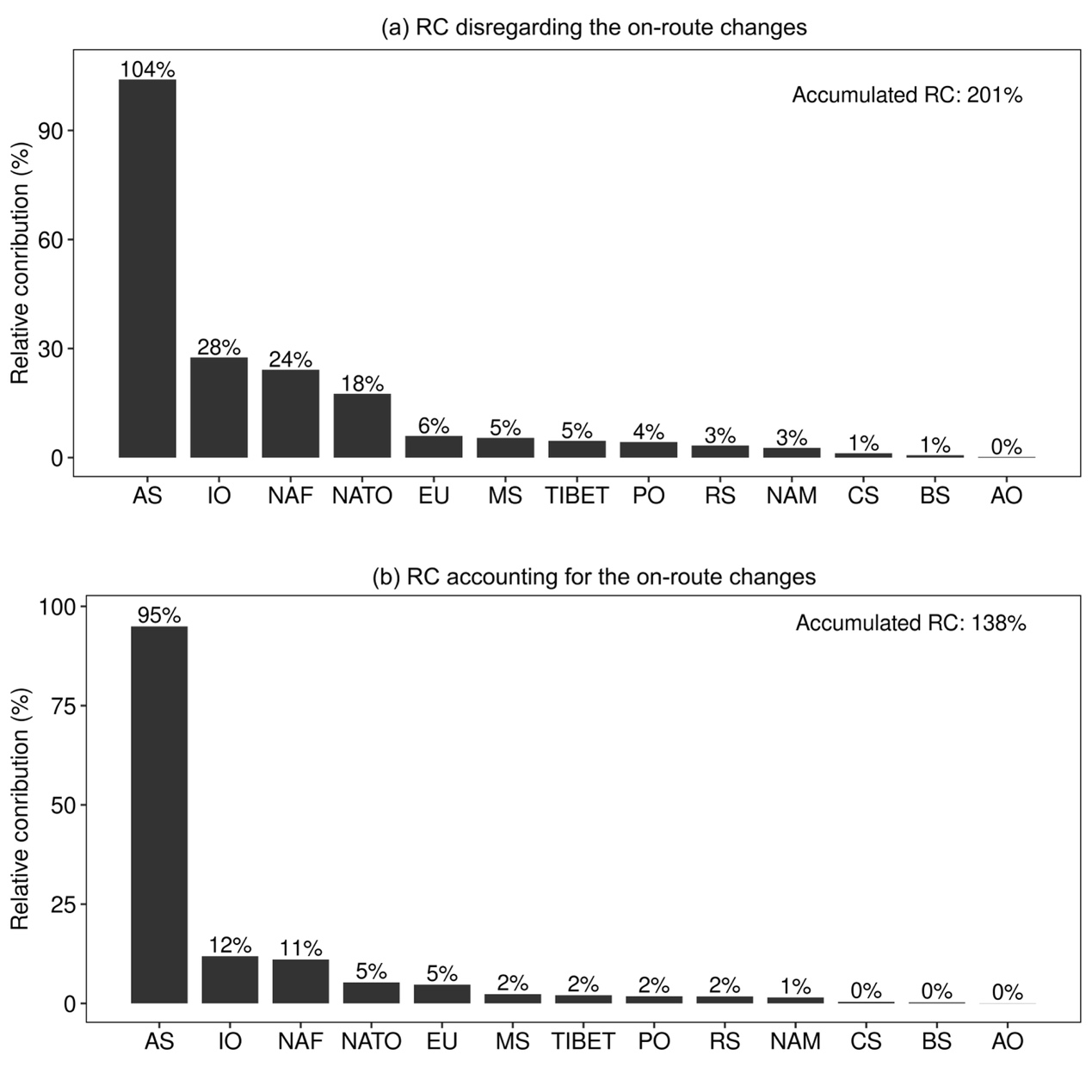


**Supplementary Figure 12. Relative contribution of water vapor based on moisture gain from source regions.** (a-b) refer to the relative contributions without (a) and with (b) considering the on-route changes. And results used to generate the figure are available in Zenodo^34^.


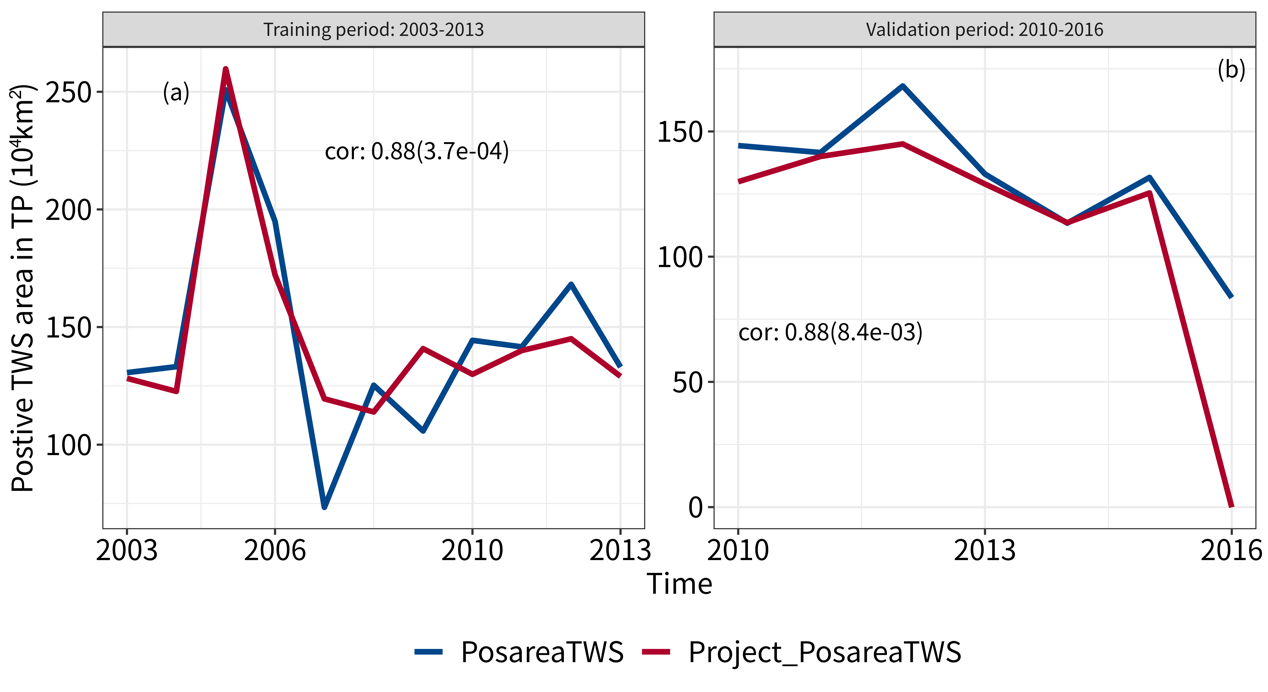


**Supplementary Figure 13. Projection model of the area under positive TWS in TP**. (a) refers to the temporal evolution in observed area (PosareaTWS) and projected area (Project_PosareaTWS) by the model, where annual sums of TWS are positive in the TP during the training period 2003-2013. (b) is same as (a) but for the validation period 2010-2016. And results used to generate the figure are available in Zenodo^34^.


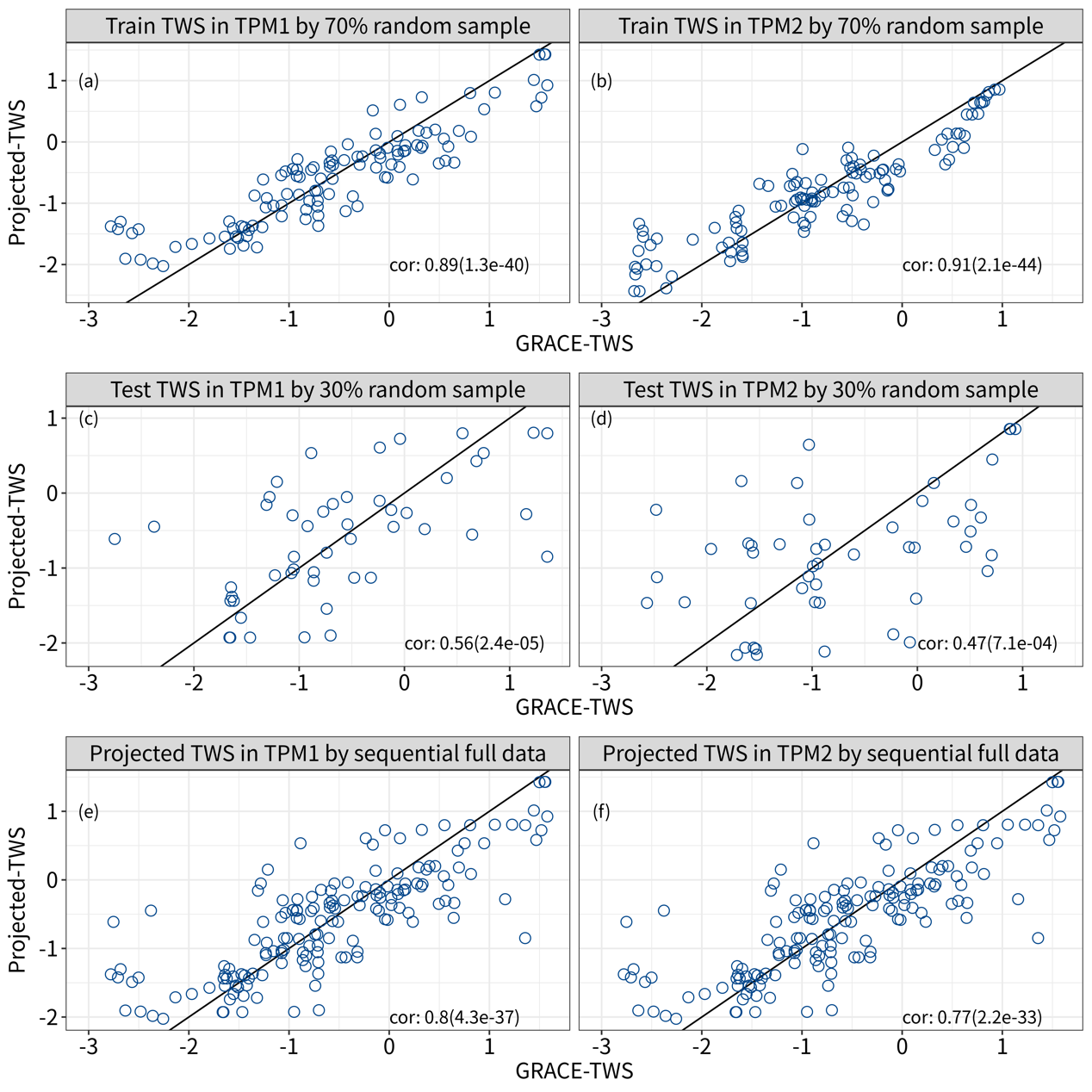


**Supplementary Figure 14.** **PME-based projection model of the TWSs in TPMs**. (a-b) refer to the comparison between observed TWS and projected TWS over the southwest (TPM1) and southeast (TPM2) TP mountains by 70% training samples. (c-d) refer to the comparison between them by 30% testing samples. (e-f) refer to the comparisons between sequential observed TWSs and projected sequential TWSs over the southwest (TPM1) and southeast (TPM2) TP mountains during 2003-2016. And results used to generate the figure are available in Zenodo^34^.


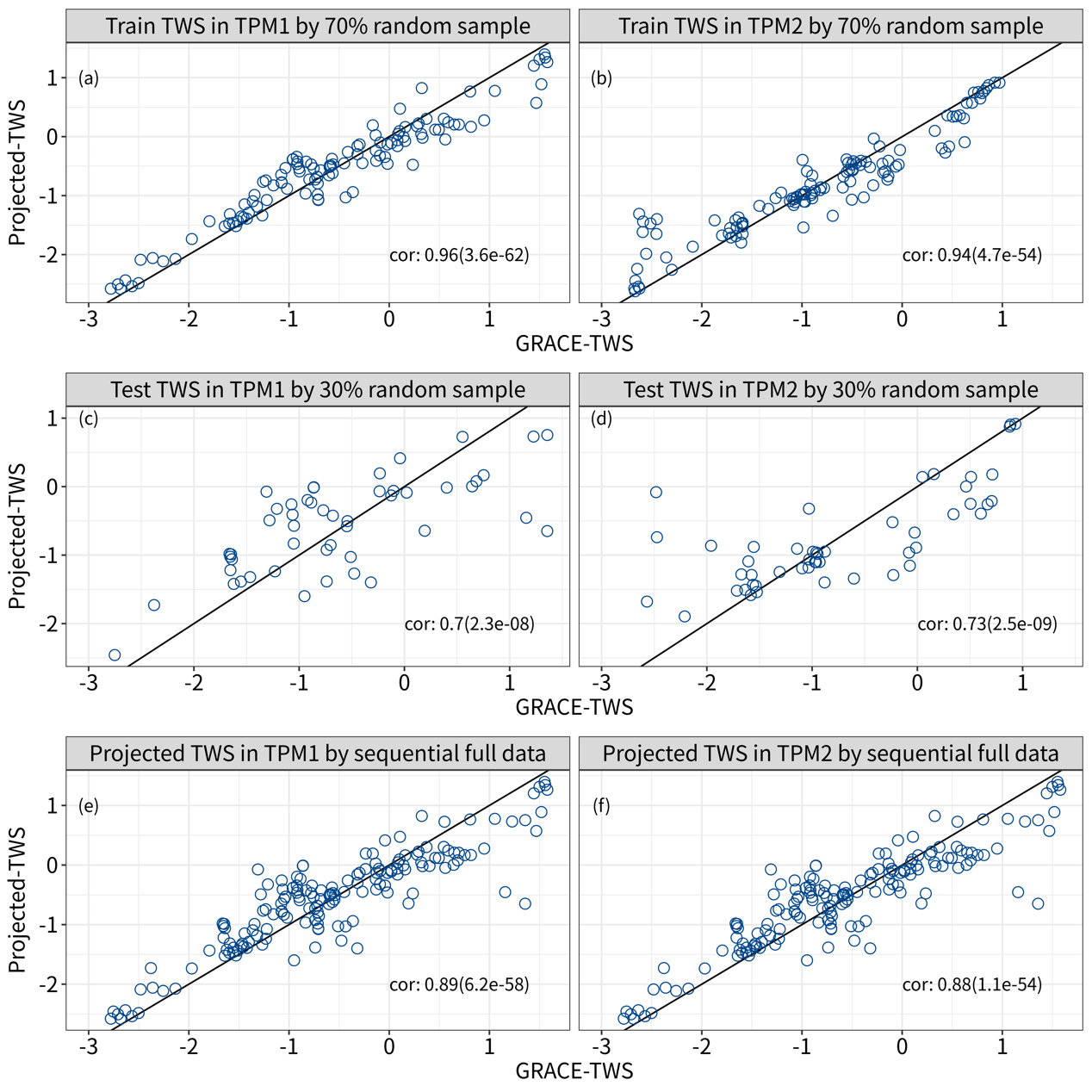


**Supplementary Figure 15. T-PME-based projection models of the TWS in TPMs**. (a-b) refer to the comparison between observed TWS and projected TWS over the southwest (TPM1) and southeast (TPM2) TP mountains by 70% training samples. (c-d) refer to the comparison between them by 30% testing samples. (e-f) refer to the comparisons between sequential observed TWSs and projected sequential TWSs over the southwest (TPM1) and southeast (TPM2) TP mountains during 2003-2016. And results used to generate the figure are available in Zenodo^34^.


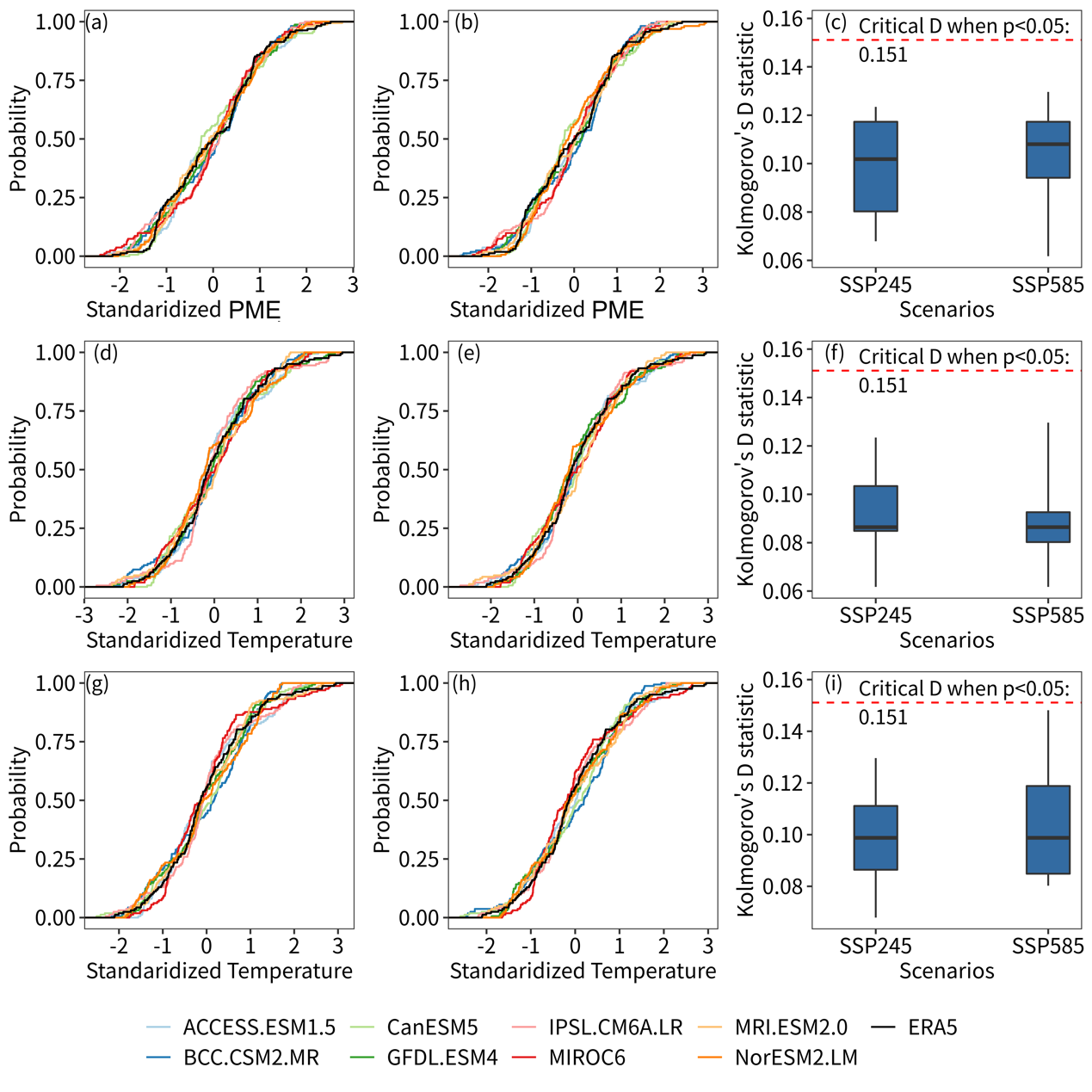


**Supplementary Figure 16. Comparison of the cumulative distributions between CMIP6 output and ERA5.** (a-c) denote the comparison of the cumulative distribution between CMIP6 and ERA5 PME over southeast North Atlantic during 2003-2016 under historical-SSP245 (a) and historical-SSP585 (b) scenarios. (d-f) denote the comparison between CMIP6 and ERA5 temperature over southwest TP mountain during 2003-2016 under historical-SSP245 (d) and historical-SSP585 (e) scenarios. (g-i) are same as (d-f) but for temperature over southeast TP mountains. Here, the Kolmogorov-Smirnov test is applied to evaluate the distance between two contributions. And results used to generate the figure are available in Zenodo^34^.


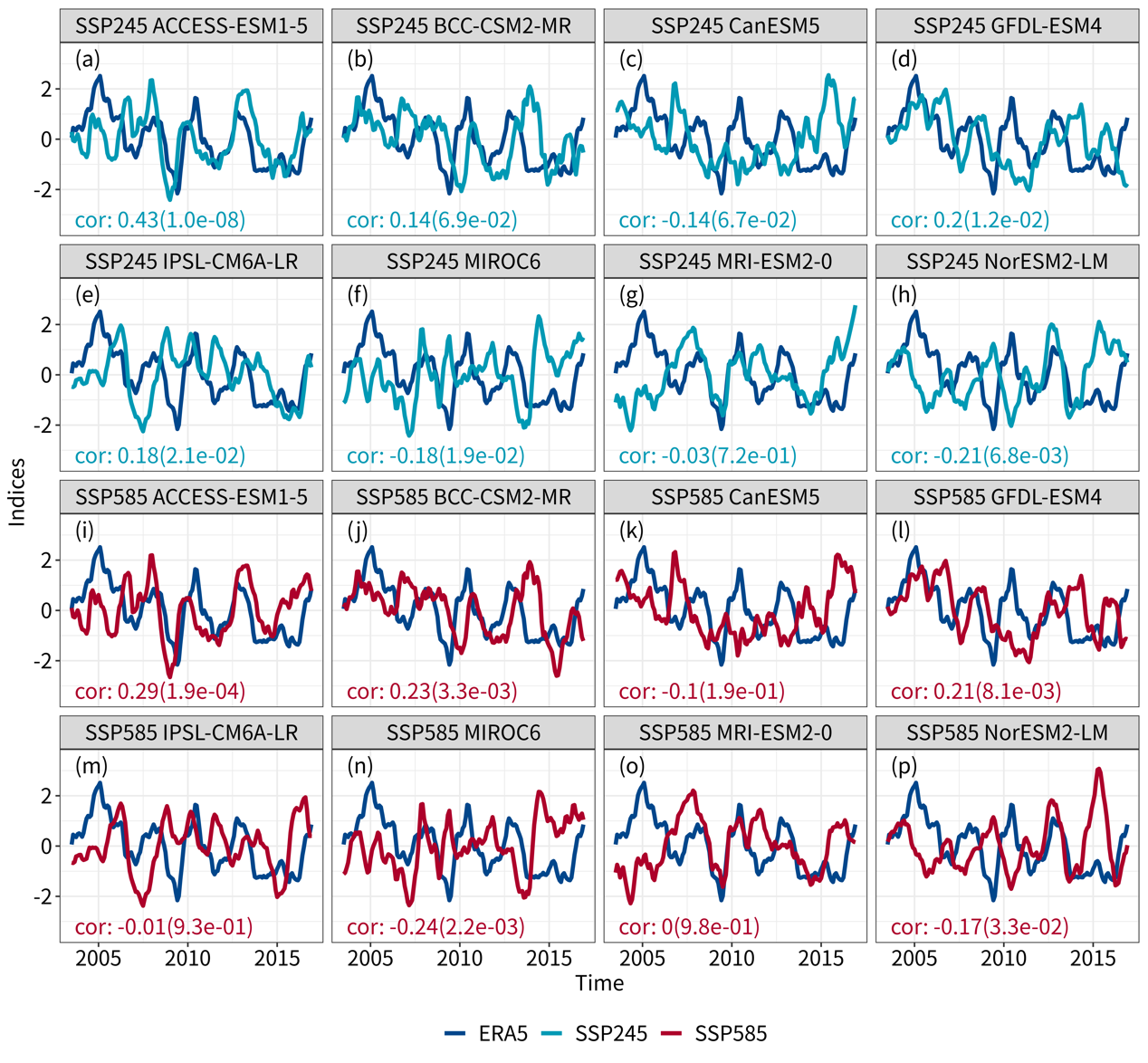


**Supplementary Figure 17. Evaluation of CMIP6 PME over the southeast North Atlantic**. Since the historical periods of the CMIP6 models all end in 2015, CMIP6-PME from 8 models during 2003-2016 under both historical-SSP245 (a-h) and historical-SSP585 (i-p) scenarios are applied to evaluate the CMIP6 PME over the southeast North Atlantic. And results used to generate the figure are available in Zenodo^34^.


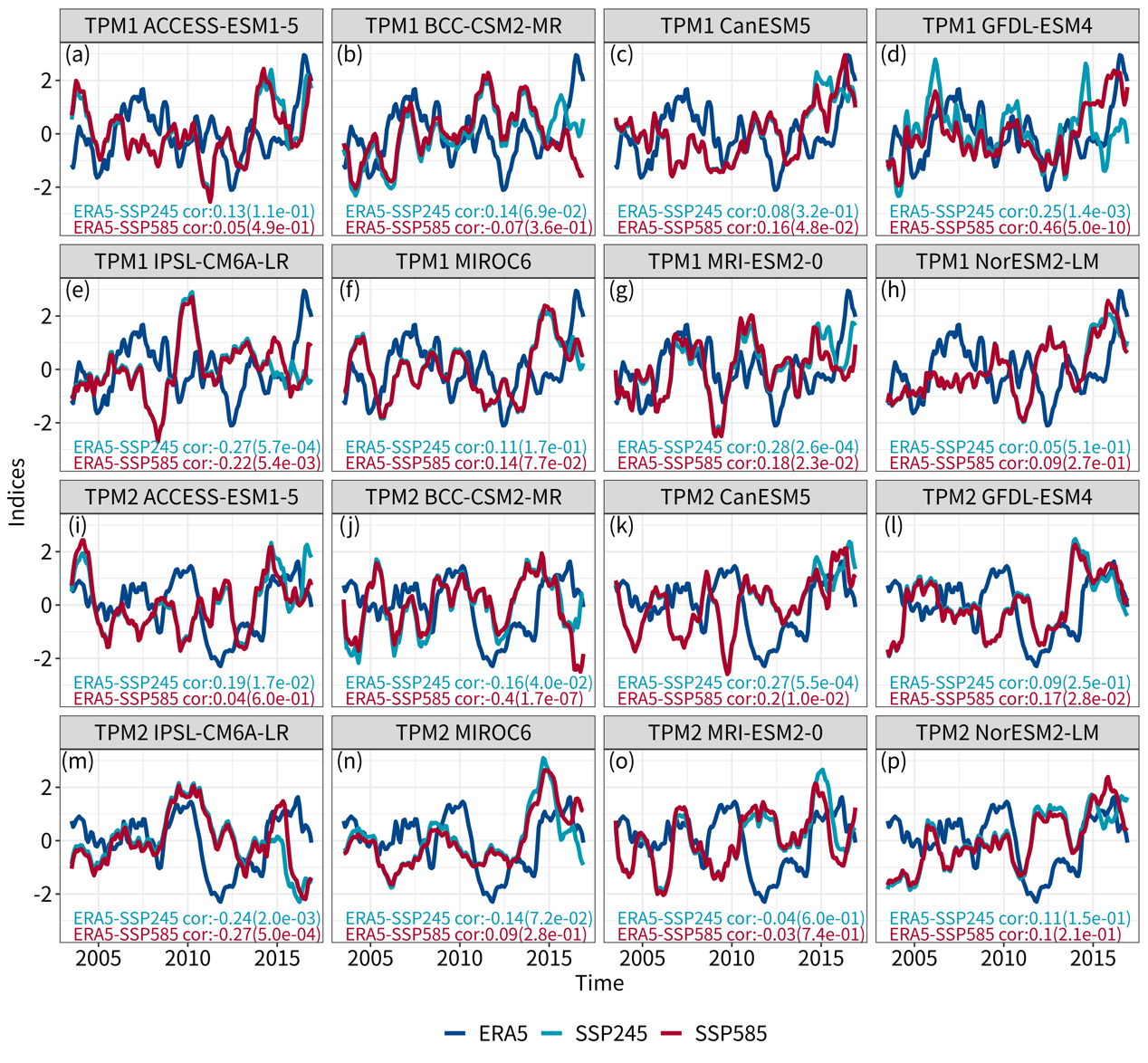


**Supplementary Figure 18. Evaluation of CMIP6 T over the southern TP mountains**. (a-h) denote evaluations of CMIP6 T over the southwest TP mountain (TPM1) under historical-SSP245 and historical-SSP585 scenarios. (i-p) denote evaluations of CMIP6 T over the southeast TP mountain (TPM2) under historical-SSP245 and historical-SSP585 scenarios. And results used to generate the figure are available in Zenodo^34^.


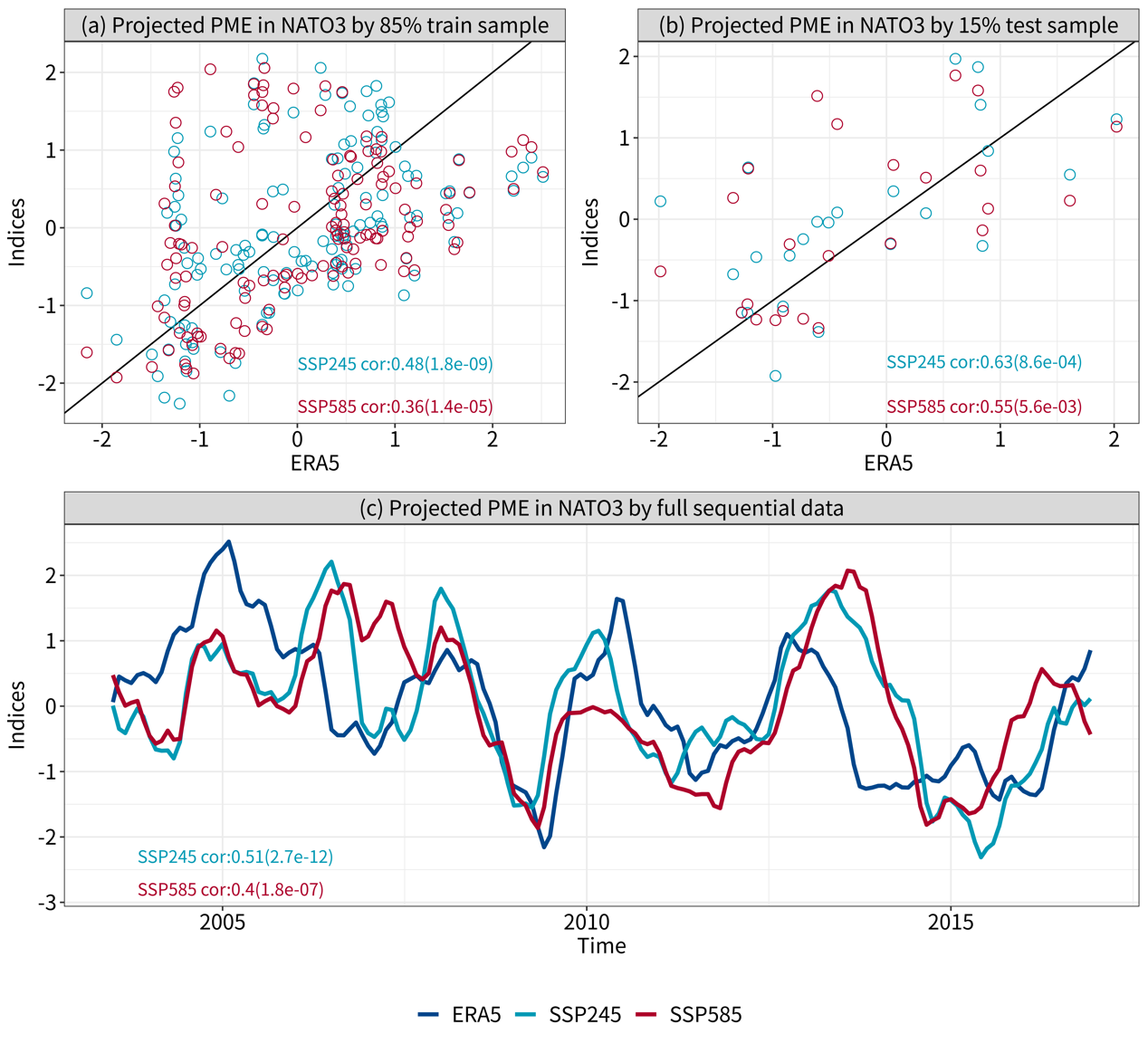


**Supplementary Figure 19.** **Evaluation of the weighting sum of CMIP6 PME ensembles in NATO3.** The whole data is randomly divided into training sample (85%) and test sample (15%). (a-b) refers to the comparison between ERA5-PME and projected weighting sum of CMIP6 PME ensembles over southeast North Atlantic (NATO3) based on 85% training samples (a) and 15% testing samples (b). The (c) refers to the temporal evolution in sequential ERA5-PME and projected sequential weighting sum of CMIP6 PME ensembles over southeast North Atlantic under historical-SSP245 and historical-SSP585 scenarios during 2003-2016. And results used to generate the figure are available in Zenodo^34^.


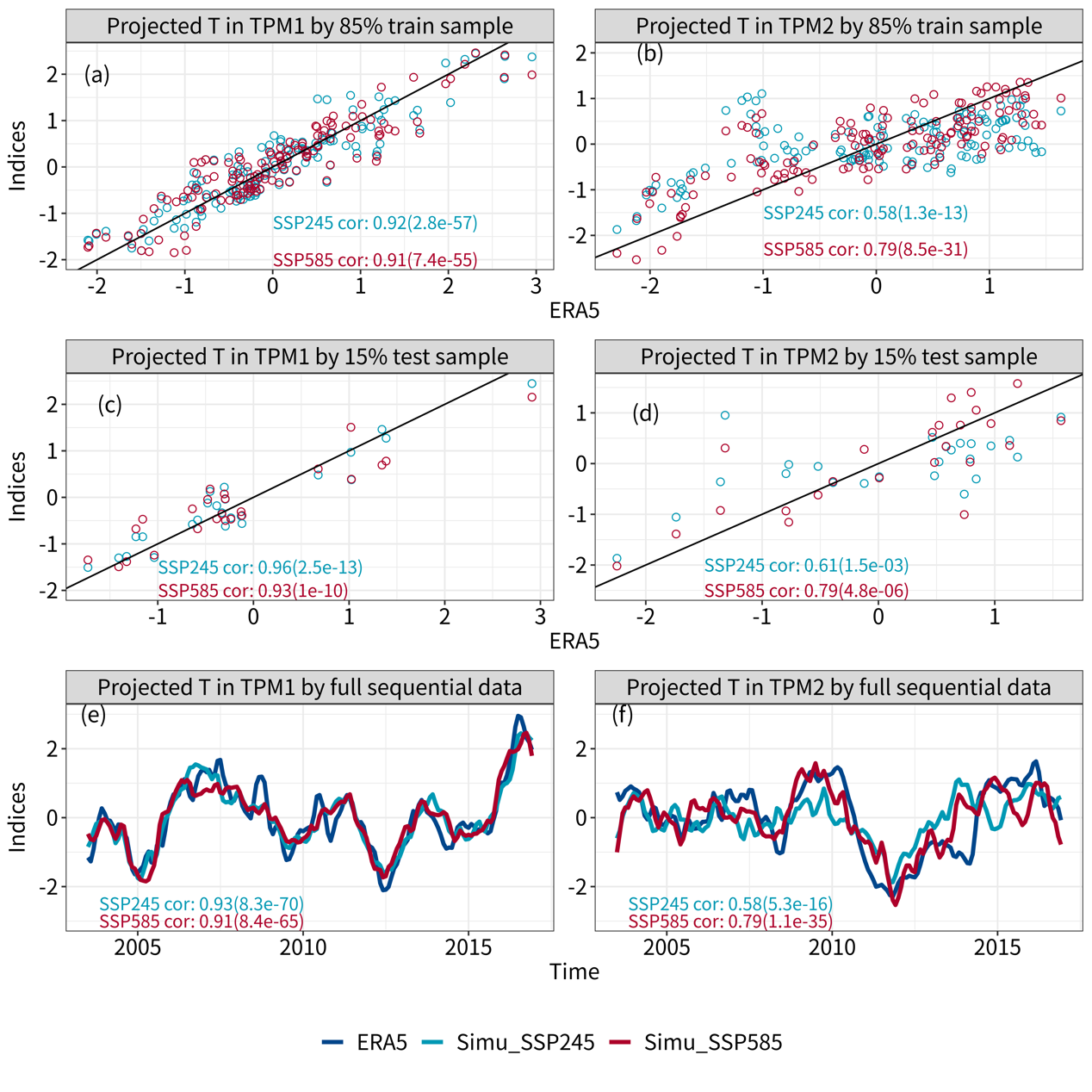


**Supplementary Figure 20.** **Evaluation of the weighting sum of CMIP6 T ensembles in TPMs.** (a-d) denote the comparison between ERA5-PME and projected weighting sum of CMIP6 PME ensembles over southwest (TPM1, a,c) and southeast (TPM2, b,d) TP mountains based on 85% training samples (a-b) and 15% testing samples (c-d). The (e-f) refer to the temporal evolution in sequential ERA5-T and projected sequential weighting sum of CMIP6 T ensembles over TPM1 (e) and TPM2 (f) under historical-SSP245 and historical-SSP585 scenarios during 2003-2016. And results used to generate the figure are available in Zenodo^34^.


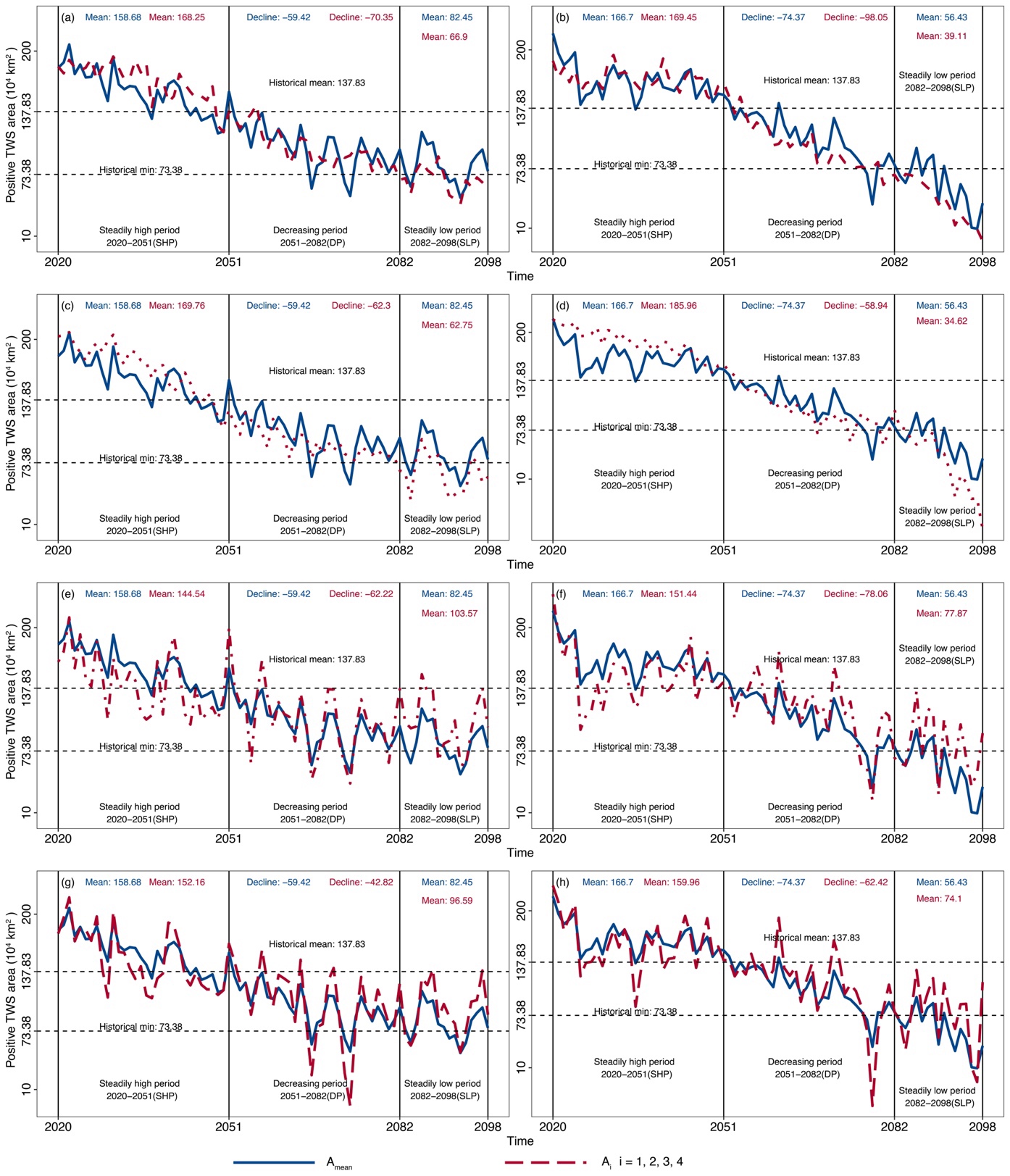


**Supplementary Figure 21. Projected area under positive TWS in TP.** (a-h) denote the projections under SSP245 (a,c,e,g) and SSP585 (b,d,f,h) scenarios during 2020-2098. Here, the $A_{mean}$ denotes the average projected area while $A_{i}$ denotes the projected area by the i^th^ input. And results used to generate the figure are available in Zenodo^34^.


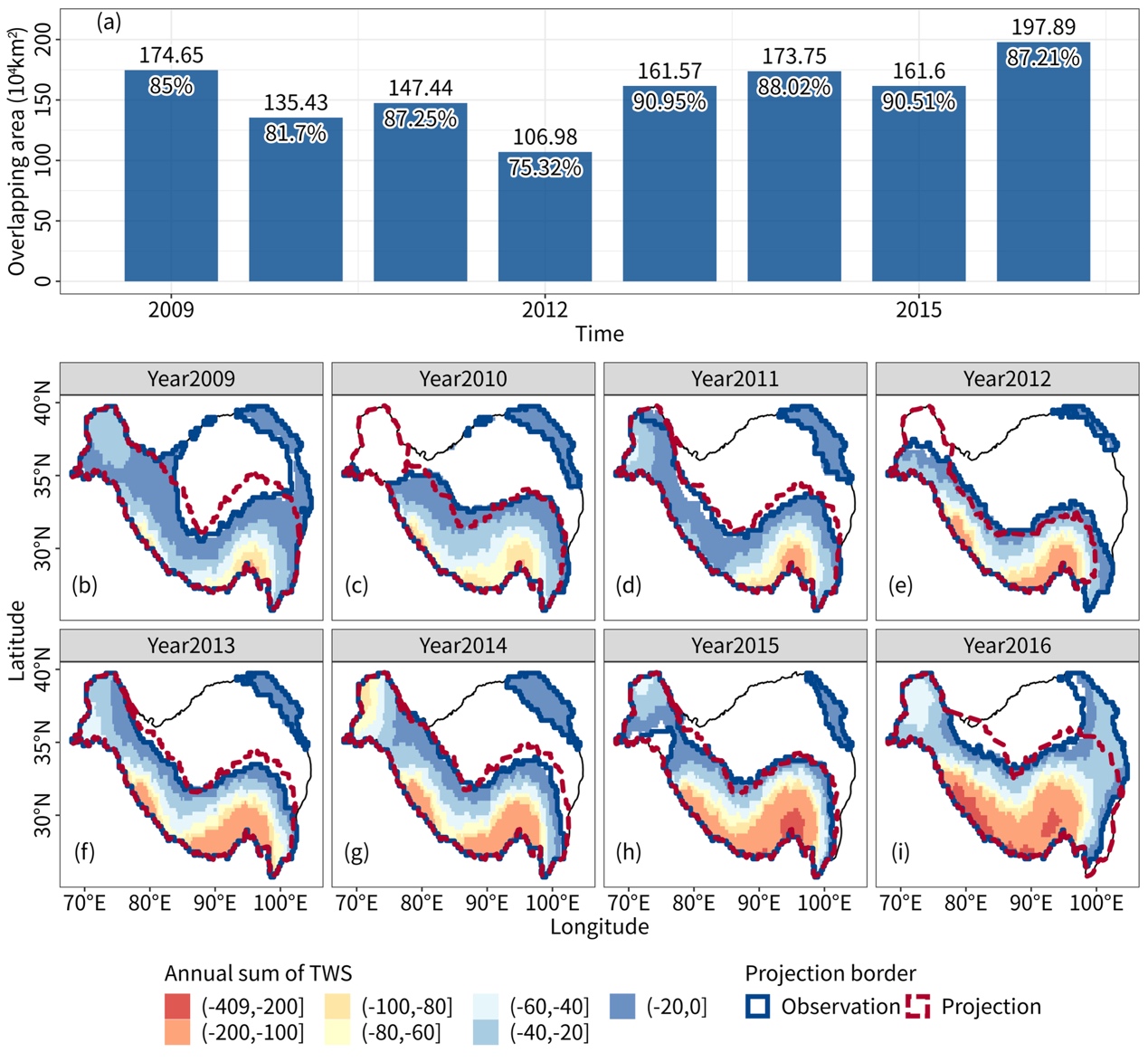


**Supplementary Figure 22. Validation of the projected northern border during 2009-2016.** (a) the overlapping areas and ratios between the observed and projected region in TP. (b-i) are the spatial comparison of the observed and projected northern borders of the region from 2009-2016. The map data of Tibet Plateau^32^ in panels **b-i** is acquired from public data source and plotted by R^33^. And results used to generate the figure are available in Zenodo^34^.


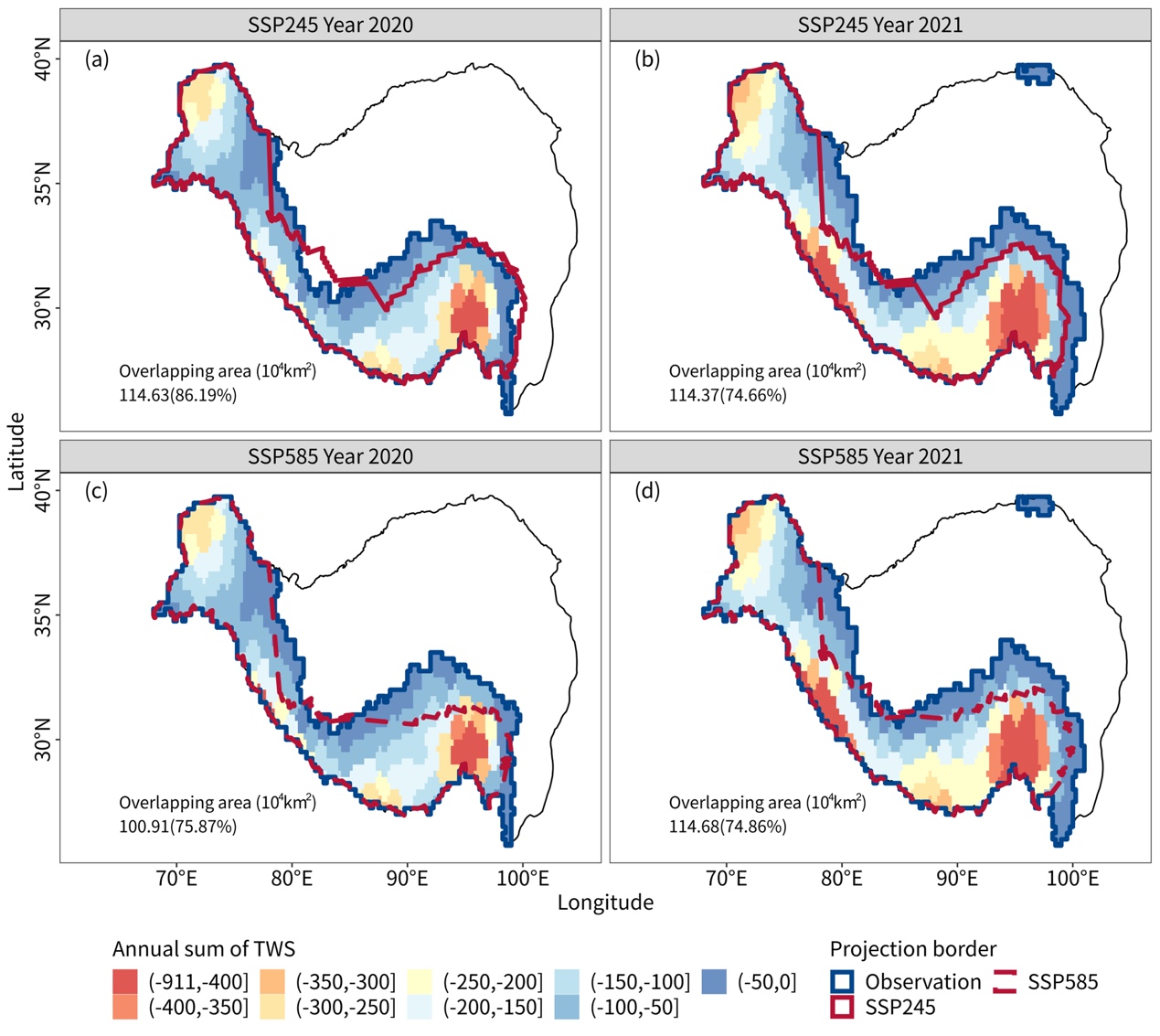


**Supplementary Figure 23. Validation of the projected northern border during 2020-2021 under SSP245 and SSP585, respectively.** (a-b) comparison of the observed and projected northern borders of the region during 2020-2021 under SSP245. (c-d) are same as (a-b) but under SSP585. The map data of Tibet Plateau^32^ in panels **a-d** is acquired from public data source and plotted by R^33^. And results used to generate the figure are available in Zenodo^34^.


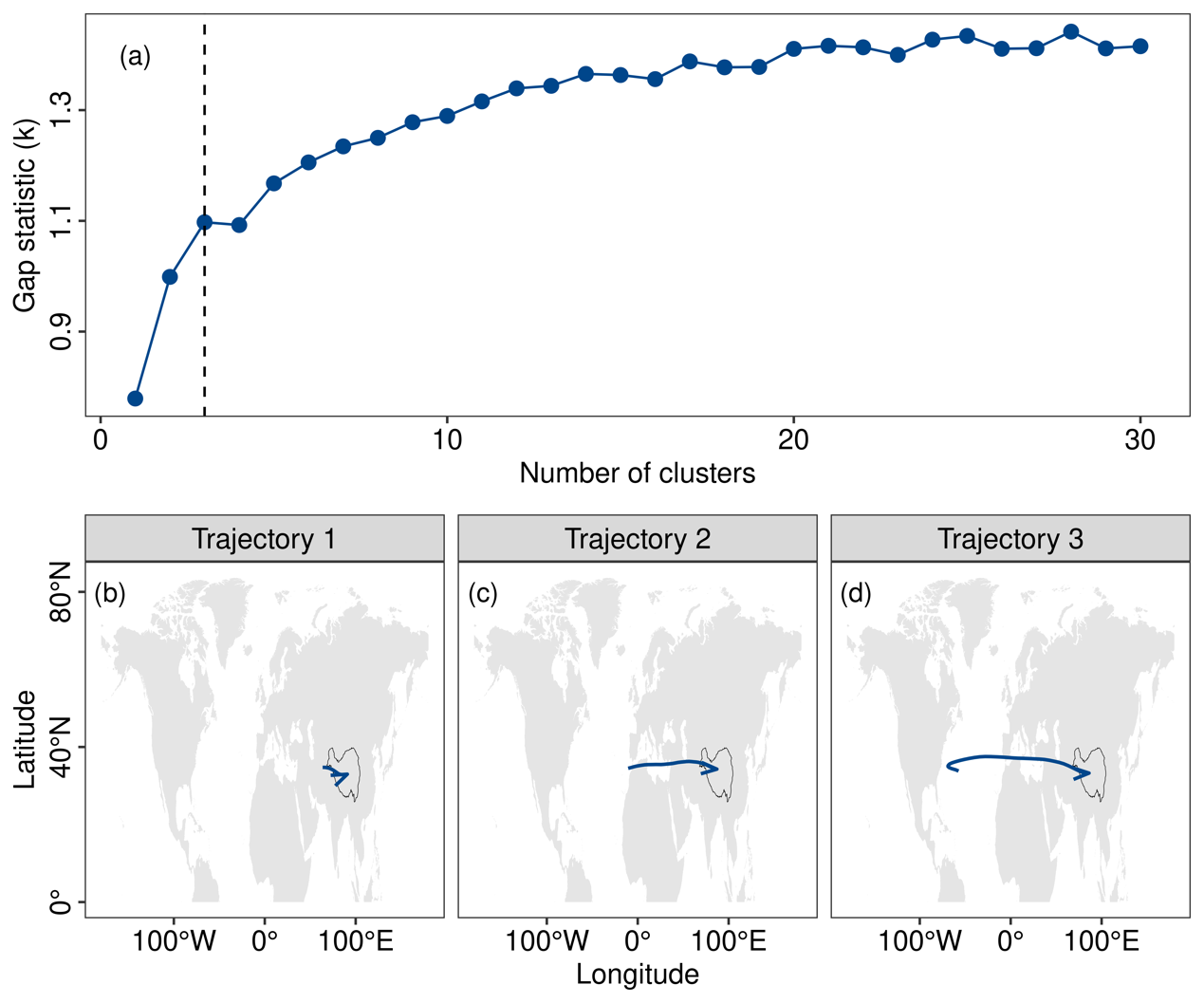


**Supplementary Figure 24. Determination of the optimum number of the clustered trajectories.** (a) is the gap statistic k value for the determination of the optimum number of the clustered trajectories. (b-d) refer to the clustered trajectories when the optimum number of the clustered trajectories is 3. The continental world map data^31^ and map data of Tibet Plateau^32^ in panels **b-d** are acquired from public data source and plotted by R^33^. And results used to generate the figure are available in Zenodo^34^.


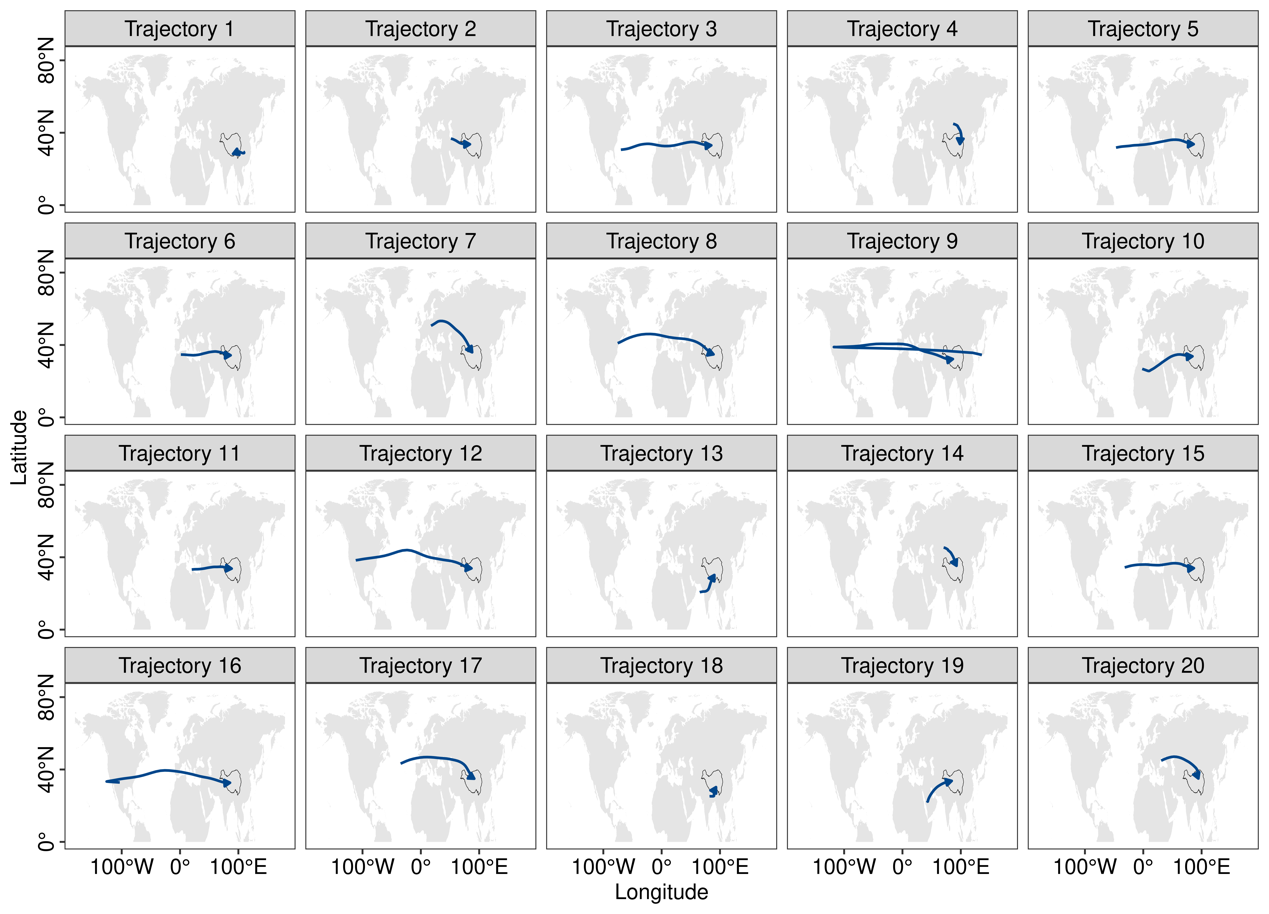


**Supplementary Figure 25. Clustered trajectories into TP.** The continental world map data^31^ and map data of Tibet Plateau^32^ in all panels are acquired from public data source and plotted by R^33^. And results used to generate the figure are available in Zenodo^34^.
